# Genetic diversity and phylogenetic relationships of *Clostridium perfringens* strains isolated from mastitis and enteritis in Egyptian dairy farms

**DOI:** 10.1186/s12866-024-03260-1

**Published:** 2024-05-06

**Authors:** Heidy Abo Elyazeed, Mahmoud Elhariri, Nashwa Ezz Eldeen, Dalal Ahmed Aziz, Rehab Elhelw

**Affiliations:** 1 Microbiology and Immunology Department, Faculty of Veterinary Medicine, Cairo University, Giza, 12211 Egypt; 2 Biology Department, Faculty of Science - Taif University, Taif, Saudi Arabia

**Keywords:** *C. perfringens*, Mastitis, Dairy, Molecular, Phylogenetic, Baby calves, Enteritis

## Abstract

**Background:**

*Clostridium perfringens*, a common environmental bacterium, is responsible for a variety of serious illnesses including food poisoning, digestive disorders, and soft tissue infections. Mastitis in lactating cattle and sudden death losses in baby calves are major problems for producers raising calves on dairy farms. The pathogenicity of this bacterium is largely mediated by its production of various toxins.

**Results:**

The study revealed that Among the examined lactating animals with a history of mastitis, diarrheal baby calves, and acute sudden death cases in calves, *C. perfringens* was isolated in 23.5% (93/395) of the total tested samples. Eighteen isolates were obtained from mastitic milk, 59 from rectal swabs, and 16 from the intestinal contents of dead calves. Most of the recovered *C. perfringens* isolates (95.6%) were identified as type A by molecular toxinotyping, except for four isolates from sudden death cases (type C). Notably, *C. perfringens* was recovered in 100% of sudden death cases compared with 32.9% of rectal swabs and 9% of milk samples. This study analyzed the phylogeny of *C. perfringens* using the plc region and identified the plc region in five Egyptian bovine isolates (milk and fecal origins). Importantly, this finding expands the known data on *C. perfringens* phospholipase C beyond reference strains in GenBank from various animal and environmental sources.

**Conclusion:**

Phylogenetic analyses of nucleotide sequence data differentiated between strains of different origins. The *plc* sequences of Egyptian *C. perfringens* strains acquired in the present study differed from those reported globally and constituted a distinct genetic ancestor.

## Introduction

*Clostridium perfringens*, a bacterial species that poses significant threats to both animal and human health, frequently contaminates food systems. This poses a risk to animals, particularly dairy herds, and can cross-contaminate ingredients and food products. Consequently, it can trigger sporadic and outbreak-related illnesses in animals and humans [1].

The ubiquitous anaerobic bacterium *C. perfringens* is found in soil, sewage, food, and feces and commonly resides in the gut of animals. This versatile pathogen causes a spectrum of illnesses in both humans and animals [2, 3].

*C. perfringens* strains can be categorized into different toxin genotypes based on the specific toxins they produce. Traditionally classified into five types (A, B, C, D, and E), the system has recently been expanded to include two new genotypes: F and G [4]. Specific *C. perfringens* toxin types are often linked to distinct illness syndromes, with the cpa-encoded alpha-toxin (phospholipase-C) found in nearly all toxinotypes [5]. and has been proposed to play a major role in both histotoxic infections, such as gas gangrene, and enteric infections, such as human food poisoning [6]. The toxin also plays a major role in several animal diseases such as enterotoxemia in calves [7] and clostridial dysentery in lambs [8, 9].

It was confirmed that type A *C. perfringens*-related bovine postpartum mortality-linked physiological stressors and gut microbiome imbalances lead to severe infections and death. This further emphasizes the potential dangers of *C. perfringens* intestinal carriage during episodes of gut microbiome dysbiosis [10]. *C. perfringens* usually does not spread directly from calf to calf in nursing systems; it is common for multiple calves in a group to be affected simultaneously due to shared exposure and management practices.

Sudden death syndrome (SDS) is a severe form of enterotoxemia that can affect multiple calves in a group simultaneously although it is not contagious. This is because calves in a group are often exposed to the same bacteria and management practices, which can lead to SDS. Once symptoms of SDS develop, treatment is difficult and mortality can occur within days [11].

Although *C. perfringens* is a major cause of clostridial enteric diseases in animals, little is known about the role of a specific type of *C. perfringens* toxin (type A) in mastitis [12].

*C. perfringens* enterotoxin (CPE) biosynthesis is associated with alpha, beta, epsilon, and iota toxins, in addition to being associated temporally with sporulation, and its synthesis begins after which most *C. perfringens* strains produce a range of toxins that induce sporulation and increase progressively with other toxins or potential virulence factors [11].

Sequencing of the alpha toxin gene in *C. perfringens* type A is crucial for unraveling the spread of *C. perfringens* infections and building effective prevention strategies. The well-documented diversity of this gene, both between and within *C. perfringens* strains, highlights the complexity of this problem. It is well established that *C. perfringens* toxin genes exhibit notable variability between different bacterial strains and even within a single strain. This variation has been reported in several previous studies [13–16].

Understanding the genetic connection between isolates recovered from different sources and their function in disease outbreaks requires the development of a direct molecular technique for the phylogenetic analysis of *C. perfringens*. In some cases, protein-coding genes, such as phospholipase C, can be a better substitute for 16 S rDNA for species differentiation [17].

This study aimed to investigate the prevalence of enterotoxigenic *C. perfringens* in Egyptian dairy cattle, and its role in mastitis, enteritis, and sudden death. Additionally, we sought to identify *C. perfringens* strains isolated from the farm chain through sequence analysis of the *C. perfringens* phospholipase C region. Our findings offer valuable insights for preventing and controlling *C. perfringens* infections in livestock, laying the groundwork for novel therapeutic strategies in future research.

## Materials and methods

### Farms

A total of 10 farms throughout Giza governorate, Cairo-Alexandria Desert Road, Fayoum, Alexandria, and Almanofia governorates were included in this study. The cattle farms were medium to large according to the production and number of lactating animals. (Small: Up to 50 cows, Medium: 20–200 cows, Large: 200–1000 cows, Very Large: Over 1000 cows) The farms produce raw milk for further processing into milk, dairy products, or both. Farms used a hazard analysis and critical control point (HACCP) approach to prevent contamination of milk and milking areas, including measures such as handwashing stations, pest control measures, and sanitization.

### Collected samples

#### Milk samples

Two hundred milk samples were collected from the lactating dairy herds. The affected animals showed signs of mastitis, may have been acutely ill from septicemia, and the udder was sometimes discolored. The affected skin area of the udder was warm to the touch and red, but the milk was usually watery. After cleaning, drying, and wiping the teat ends with 70% ethanol, the first three to four streams of milk were discarded. Udder quarter milk samples were collected aseptically from the clinically affected glands in 50-ml sterile plastic tubes.

#### Fecal samples

One hundred and fifty-nine fecal swabs were collected from animals showing symptoms of enterotoxemia, including lethargy, colic, bruxism, and fluid distension of the abomasum, with significant signs of tympany and colic preceding diarrhea, which is usually low in volume and may be fatal. Samples were collected using a sterile swab stick. The swab was handed to her caretaker, labeled, and transported to the laboratory.

#### Intestinal content samples

**Fig. 1:**
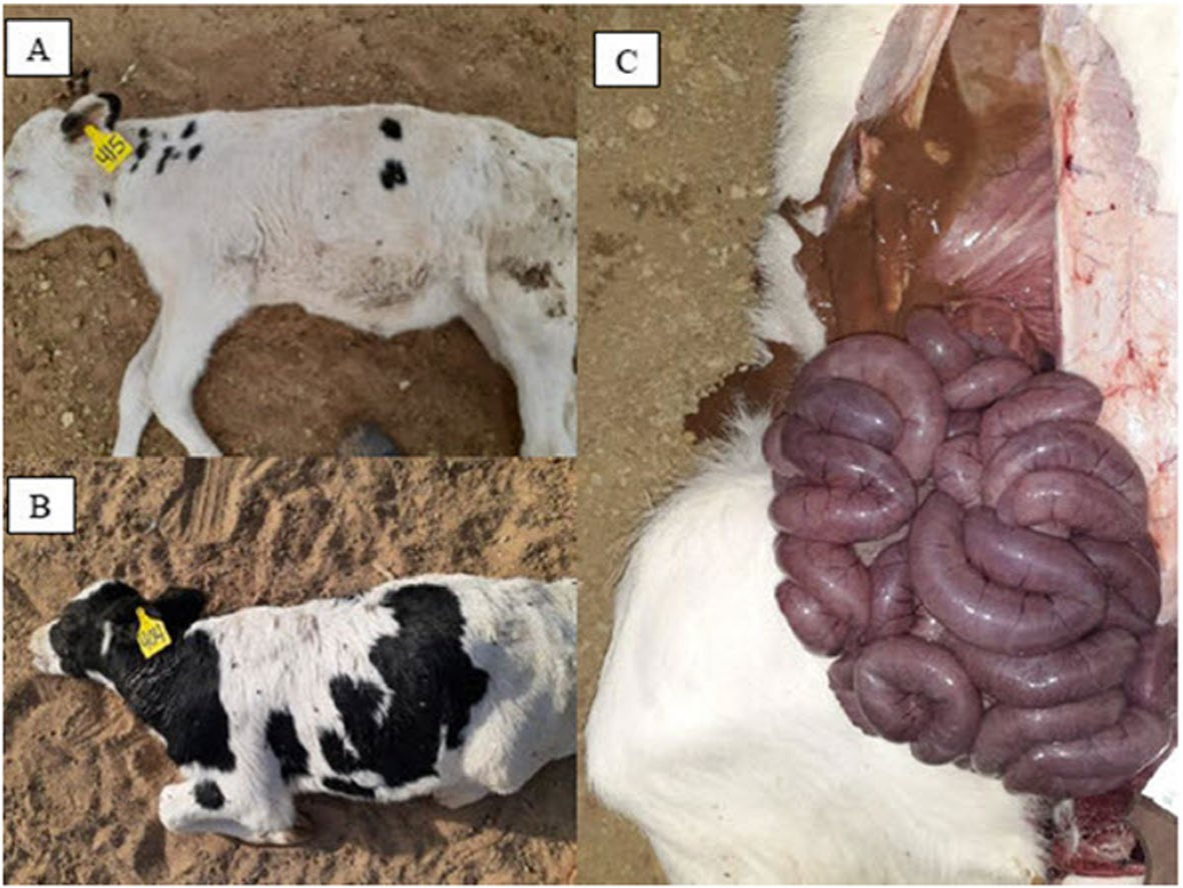
**A** & **B** Case of peracute death of Friesian newly born calf with necro-haemorrhagic enteritis from clostridia vaccinated dam (**C**) Severely dilated and congested small intestine of a case of bovine necro-haemorrhagic enteritis a typical form of sausage like appearance

Sixteen small intestinal contents were collected from calves that died unexpectedly in the first week of life, and no warning symptoms were reported by the producer before the calves were discovered. The autopsy revealed segmental intestinal hemorrhage in all calves examined, along with a significant amount of crimson fluid and clotted blood in the small intestine lumen (Fig. 1).

### Bacterial isolates, growth conditions and biochemical identification

The milk samples obtained were centrifuged for 20 min at 3,000 rpm. The sediments of each milk sample were added to cooked meat broth (CMB) after removing the cream and supernatant. The samples were incubated anaerobically according to [18]. A loopful from each sample was streaked onto sheep blood agar plates containing 150 µg/ml neomycin sulfate and incubated anaerobically at 37 °C for a further 24 h after overnight incubation using anaerobic jars containing 95% H2 and 5% CO2 (AnaeroGen, OXOID, Ltd, England). To eliminate non-spore-forming bacteria, sterile swabs from diarrheal fecal samples were diluted in PBS (1:10) and grown in CMB at 80 °C in a water bath for 5 min. The CMB tubes were then incubated anaerobically at 37 °C the next day in a jar with gas production kits and then transferred to sheep blood agar with neomycin. C. perfringens has been distinguished from other Clostridium spp. by biochemical screening tests, including oxidase, catalase, motility nitrate reduction, blood hemolysis, indole production, urea hydrolysis, lecithinase, and sugar fermentation tests [19].

### *C. perfringens* DNA extraction and toxin genotyping

A starter culture (5 ml) of each *C. perfringens* isolate was prepared by overnight growth at 37 °C in tryptic soya broth. The bacterial broth was centrifuged, washed with TE buffer, and prepared for genomic DNA extraction using a Genomic DNA–Spin column (Jena Bioscience, Germany) according to the manufacturer’s instructions. Toxin gene primers for the alpha, beta, iota, and epsilon subtypes of *C. perfringens* were detected using a multiplex polymerase chain reaction assay (Table 1) according to [20]. DNA samples were amplified in 25 µl of the following reaction mixture: 5 µl of DNA as template, 0.34 mM of each cpe oligo, 0.36 mM of each cpb oligo, 0.44 mM of each etx oligo, 0.5 mM of each cpa oligo, 0.52 mM of each iA oligo, 12.5 µl of EmeraldAmp Max PCR Master Mix (Takara, Japan), and completed to 25 µl by DNase-RNase-free water. The PCR cycling program was performed in the thermal cycler as follows: the amplification condition for toxins was performed with initial denaturation at 94 °C for 3 min, 35 cycles of denaturation at 94 °C for 1 min, primer annealing at 55 °C for 1 min. Primer extension was performed at 72 °C for 1 min, followed by a final extension at 72 °C for 10 min. Subsequently, the PCR products were analyzed by applying 20 µL to a 1.5% agarose gel for electrophoresis and visualized with ethidium bromide on an ultraviolet transilluminator. The negative and positive control strains used in this study, *C. perfringens* type A (ATCC 13,124), B (ATCC 3626), (ATCC 10,543), and D (NCTC 8346), were used as controls for the different toxins.) Experiments were performed using all reagents except template DNA [20].

**Table 1:** Primers for the five toxins genes of *C. perfringens* used in multiplex PCR

Toxin/gene primer	Nucleotide sequence 5 ^`^to 3 ^`^	Amplicon (bp)
***cpa***		324
**Forward**	GCTAATGTTACTGCCGTTGA	
**Reverse**	CCTCTGATACATCGTGTAAG**`**	
***cpb***		196
**Forward**	GCGAATATGCTGAATCATCTA	
**Reverse**	GCAGGAACATTAGTATATCTTC`	
***etx***		655
**Forward**	GCGGTGATATCCATCTATTC	
**Reverse**	CCACTTACTTGTCCTACTAAC	
***iap***		446
**Forward**	ACTACTCTCAGACAAGACAG	
**Reverse**	CTTTCCTTCTATTACTATACG`	
***cpe***		233^a^
**Forward**	GGAGATGGTTGGATATTAGG	
**Reverse**	GGACCAGCAGTTGTAGATA	
***plc***		
** Plc-MHF1**	AAAATTAACGGGGGATATAAAAATGAAAAG	
**Plc-MHF2**	AGAGCAGGTAAGGTTAGATGTGTTTAATTA	1259^b^
**Plc-MHR3**	GTAAATACCACCAAAACCAAT	1560^b^

^a^[20, 21]

^b^The present study

### A novel oligonucleotide primer designed for phospholipase C 

The primer was designed to identify *C. perfringens* phospholipase C based on published amino acid sequences available in the UniProt database. Primer designed for amplifying the complete coding domain sequence, as the previous published primer for *plc* gene amplified only parts of the respective open reading frames. The present study primers plc-MHF and plc-MHR were designed to target the *plc* gene (Table 1). The gene sequence was obtained from the NCBI GenBank database (http://www.ncbi.nlm.nih.gov/genbank/). A multiple alignment of the gene sequences was carried out utilizing the nucleotide blast (https://blast.ncbi.nlm.nih.gov/Blast.cgi) In Silco analysis of *plc* gene sequences from 14 *C. perfringens* strains, including the three reference strains ATCC 13,124, 8346, 10,543, and NCTC 3626 (Table 2). In silico analysis identified *C. perfringens* plc-specific target sites and a potential primer set for detection using https://www.ncbi.nlm.nih.gov/tools/primer-blast/index.cgi.We verified the primer’s specificity by searching against the BLAST database to minimize the possibility of amplifying non-target sequences by Basic Local Alignment Search Tool) database search application (http://www.ncbi.nlm.nih.gov/BLAST) Table 2. The study referenced a previously published primer pair [21] to amplify a 324-base pair (bp) fragment of the *cpa* gene located on the chromosomal *cpa* locus. PCR mix solution was prepared as Master Mix 25 (Dream Taq), DNA template 5µL, Primer pair 2 µL. Nuclease-free water (up to 50 µL). PCR conditions were as follows: initial denaturation at 94 °C for 5 min, denaturation at 94 °C for 1 min, annealing at 60 °C for 1 min, extension at 72 °C for 1 min, and a final extension at 72 °C for 7 min. Amplicons of phospholipase C were separated using 1% agarose gel electrophoresis.

**Table 2:** Results of testing the specificity of primers designed for *plc* and *cpa* targed genes

Taxon	Strain	Accession no.	Primers set reaction	
			**PLC-MH-F/R** **(*plc*-target)**	**(*cpa*-target)**
*C. perfringens*	ATCC 13,124		+	+
*C. perfringens*	ATCC 8346		-	+
*C. perfringens*	ATCC 10,543		-	+
*C. perfringens*	ATCC 3626		-	+
*C. perfringens*	L9	D49968.1	+	+
*C. perfringens*		D63911.1	+	+
*C. perfringens*	CPA19	OR626596.1	+	+
*C. perfringens*	Saigas/2013	KP143661.1	+	+
*C. perfringens*	Saigas/2012	KP143660.1	+	+
*C. perfringens*	NCIB1063	D49969.1	+	+
*C. perfringens*	KZ211	D32124.1	+	+
*C. perfringens*	PB6KN5L7	D32123.1	+	+
*C. perfringens*	ORF2	D10248.1	+	+
*C. perfringens*		L43546.1	+	+

### Molecular identification of *plc* gene

Five positive DNA bands with targeted sizes were eluted and purified using a gel extraction kit (QIAquick, Qiagen). A BigDye Terminator 3.1 Cycle Sequencing Kit was used (Applied Biosystems, USA). Sequencing reactions were further purified using the Centri-Sep Purification Kit (Applied Biosystems) and decoded using a 3500 Genetic Analyzer (Applied Biosystems). Five *C. perfringens* strains (one from milk and the other from feces) were identified by sequence analysis of plc region sequencing. Homologies between nucleotide sequences of the detected phospholipase C gene and others published in GenBank were determined using BLAST 2.0 search programs (National Centre for Biotechnology Information, ‘NCBI’; http://www.ncbi.nlm.nih.gov/). The BLAST search assigns scores to matches with a clear statistical meaning, making it easier to identify real matches from random hits [22].

#### Sequencing and phylogeny tree construction

The BioEdit Sequence Alignment Editor 7.2.5 [23] was used to prepare sequence alignments. To ascertain their phylogenetic relationship, the partial sequences obtained from *C. perfringens* isolates were first compared with reference sequences using BLAST (National Center for Biotechnology Information at www.ncbi.nlm.gov/BLAST). The most closely related reference strains were obtained from the GenBank database. The highest-scoring database sequence was retrieved in aligned form from the GenBank data and aligned with the sequences of the current investigation for all sequences with database relatives that demonstrated a similarity value. In addition, GenBank sequences for a few key bacterial groups were obtained. MEGA11 software was used to create a UPGMA (Completed CDS) & Maximum Parsimony (Partial CDS) phylogenetic tree with 1000 bootstrap replicates to verify the tree [24]. Comparative analysis of sequences and similarity matrices was performed using the CLUSTALW multiple sequence alignment program, version 7.1 of the MegAlign suite of Lasergene DNASTAR software [25], to determine nucleotide and amino acid sequence similarities and relationships.

### Nucleotide sequence accession number

The *plc* region sequence was determined for the five bacterial strains, and uploaded to the GenBank database (http://www.ncbi.nlm.nih.gov) is available under the following accession numbers: KX524150 (cow milk), KX524151 (baby calf feces, sudden death), MN635790, MN635792 (Fecal), and PP002324 (baby calves, intestinal content).

## Results

### Prevalence of *C. perfringens* among the examined samples

**Fig. 2:**
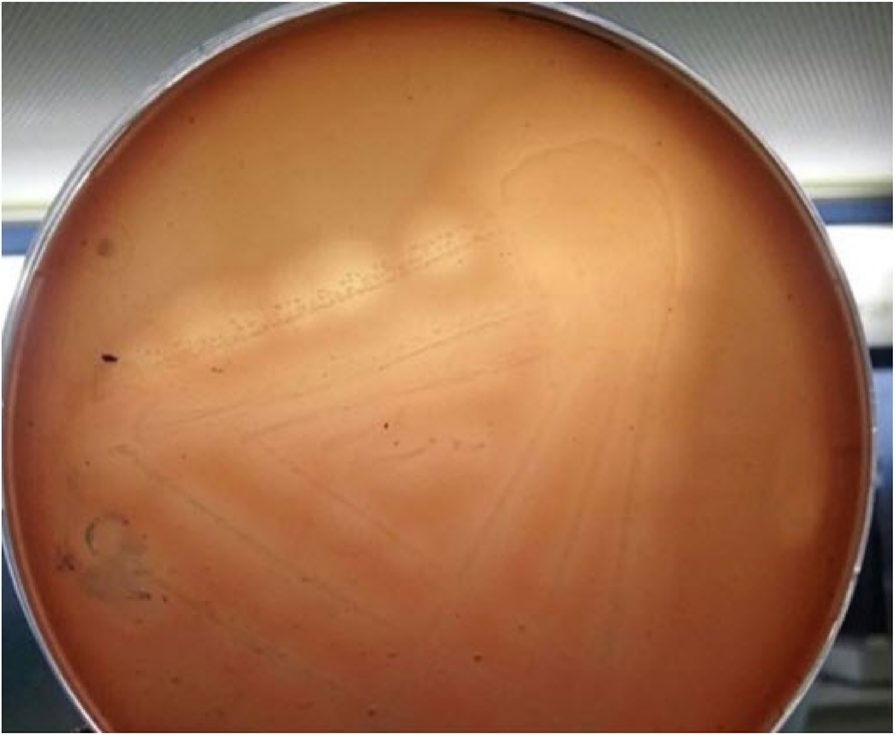
A close-up macroscopic characteristics feature of *C. perfringens* double zones hemolysis on sheep blood agar with neomycin

**Fig. 3:**
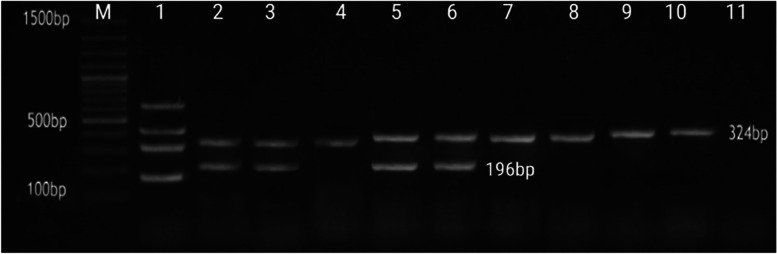
Agarose gel electrophoresis of multiplex PCR showing amplification of a 324 bp fragment of Alpha-toxin gene and 196 of Beta-toxin gene from the extracted DNA of *C. perfringens* isolates, Lane (M) DNA marker GeneRuler 100 bp plus (Thermofisher), Lane (1) positive control, lane (11) negative control

The recovery rate of *C. perfringens* was determined according to its growth characteristics, as it produced a double zone of hemolysis on 10% neomycin sheep blood agar, as shown in Fig. (2). Colonies of *C. perfringens* appear flat and olive-colored. All suspected colonies were gram-positive short-plumb bacilli, rarely having central oval non-bulging endospores, and were further identified by standard biochemical reactions. As shown in Table (3), 93 *C. perfringens* isolates were recovered from the total examined samples (395), with a detection rate of 23.5%. *C. perfringens* was isolated from milk samples of mastitic cases (18/200), rectal swabs of diarrheal cases (59/179), and sudden death cases (16/16) with an occurance of 9%, 32.9, and 32.9% 100, respectively Fig. (3).

**Table 3:** Recovery rate of *C. perfringens* among the examined samples

Farm code	Mastitic lactating cow no.	*C. perfringens*	Diarrheic baby calves no.	*C. perfringens*	Sudden death baby calves	*C. perfringens*
I	20	2	10	4	1	1
II	35	2	30	4	2	2
III	15	1	12	5	1	1
IV	20	1	18	2	-	-
V	10	3	8	6	-	-
VI	40	1	33	12	5	5
VII	20	2	15	8	-	-
VIII	10	1	7	5	-	-
IX	25	2	21	6	4	4
X	25	3	25	7	3	3
Total	200	18 (9%)	179	59 (32.9%)	16	16 (100%)

 According to toxin genotyping, all of the isolates (93 (100%) harbored the alpha toxin cpa+, the marker toxin for *C. perfringens*, all of which were enterotoxigenic strains (*cpe*^+ve^); thus, most of the strains were type A (95.6%), whereas the four strains of sudden death cases (*cpe*^+ve^) and (*cpb*^+ve^) (4.3%) were type C (Tables 3 and 4).

**Table 4:** Recovery rate of *plc *and *cpe *genes of *C. perfringens* strains from different sources by using multiplex PCR technique

Strain source	Number of strains tested	Number (%) of that strains were				
		***cpa***^**+**^	***cpb***^**+**^	***etx***^**+**^	***iap***^**+**^	***cpe***^**+**^
Milk	18	18	0	0	0	18
Rectal swabs	59	59	0	0	0	59
Intestinal content	16	16	4	0	0	16
Total	93	93 (100%)	4 (4.3%)	0	0	89 (95.6%)

### Amplification and sequencing of phospholipase C gene using a newly designed oligonucleotide primer

**Fig. 4:**
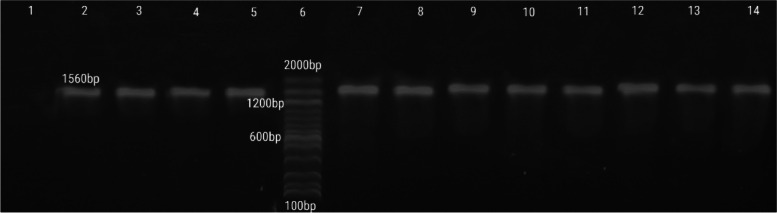
Agarose gel electrophoresis showing amplification of a 1560 bp phospholipase C gene (*plc*) from the extracted DNA of *C. perfringen*s isolates, Lane 6 DNA ladder 100 bp plus (Invitrogen), Lane 1 negative control, Lane 2 positive control

For the full analysis of phospholipase C (*plc*), a new PCR primer was used for the full sequence analysis of the gene, especially in the five selected isolates, which were representative of all clinical cases in the present study of *C. perfringens* from milk, diarrhea, and intestinal content of calves with sudden death. The isolates were tested with a new primer, in addition to the positive control. The *plc* gene was amplified in all tested strains with an amplicon of an average size of 1560 bp (Fig. 4).

The results, presented in Table 2, demonstrate; the high specificity of the *plc* new primer set. This primer set amplified only *C. perfringens plc* DNA, confirming its ability to detect the bacterium at the species level. No amplification occurred with any non-target strains, validating its design accuracy. Confirming the specificity of the *plc-*target gene was applied by conventional PCR by the *cpa* primer set. This primer set only amplified DNA from strains known to contain a functional chromosomal *cpa* gene (Table 2).

#### Phylogenetic analysis of phospholipase C complete CDS

The *plc* sequence analysis of five *C. perfringens* isolates was performed on milk, three diarrheal cases, and intestinal contents of one dead case. The complete open reading frame for *plc* gene were applied in three strains.

A total of 9 referral sequences from α-toxins were analyzed. The alignments and comparisons of the *C. perfringens* sequences of alpha toxins obtained in the present study, and the other ten referral strains from the GenBank database. According to the query and matching sequences on GenBank according to the BLAST output, the nucleotide sequences of the selected phospholipase C amino acid sequences share main regions of identity with other queries of different referral *C. perfringens* strains on GenBank.

**Fig. 5:**
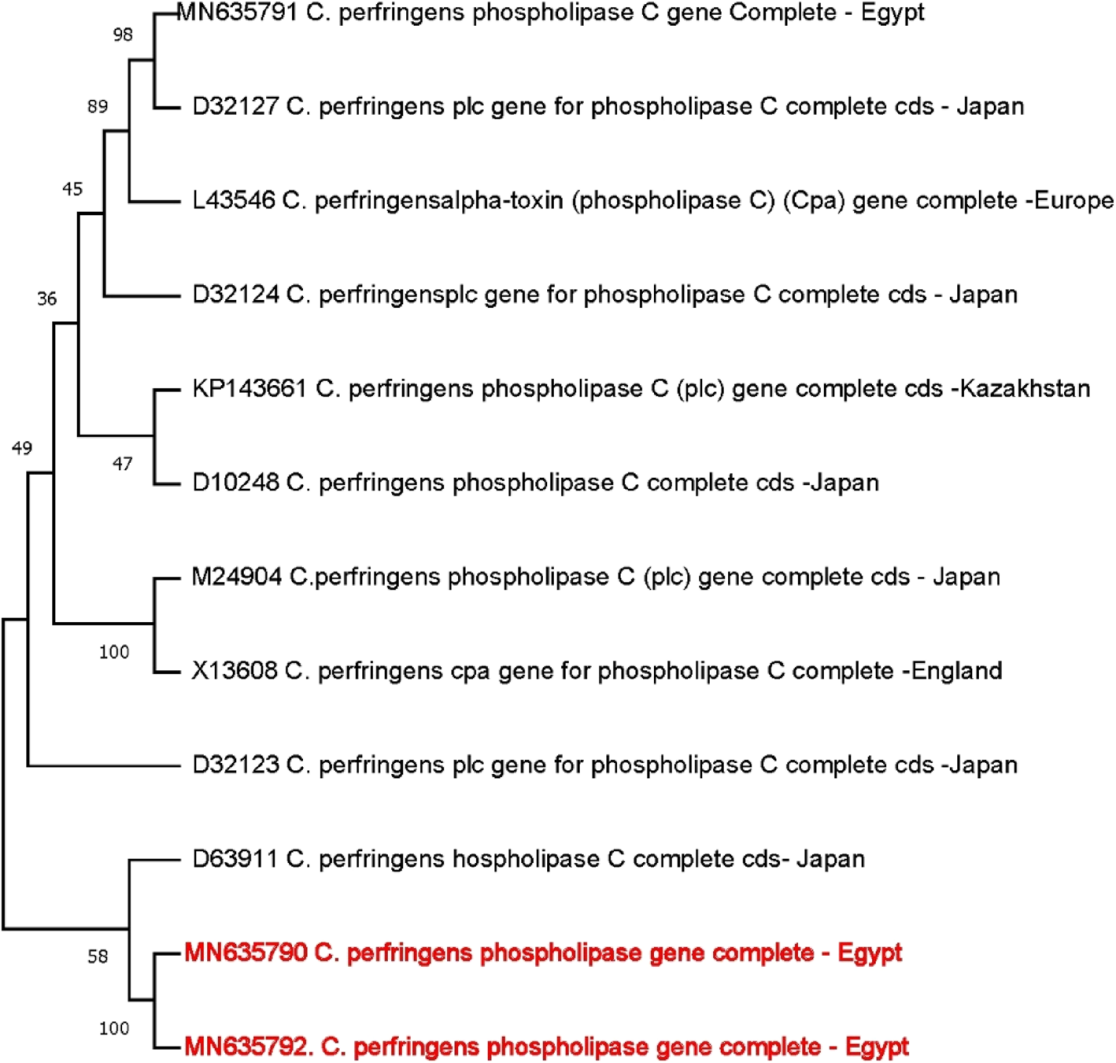
The evolutionary tree of the isolated *C. perfringens* phospholipase C (*plc*) open reading frame and the obtained 10 referral nucleotide sequences from GenBank. The tree was constructed by using the UPGMA method. The bootstrap consensus tree inferred from 1000 replicates is taken to represent the evolutionary history of the taxa analyzed. The evolutionary distances were computed using the JTT matrix-based method. Evolutionary analyses were conducted in MEGA11

A UPGMA evolutionary tree depicting the entire coding region of the *plc* gene is presented in Fig. 5. The Egyptian strains MN635790 and MN635792 were used as the outgroup to root the tree. Bootstrap analysis with 1,000 computer-generated trees determined the reliability of the branching order. The sequences of twelve clostridial species formed a single evolutionary unit (monophyletic group). Two Egyptian *C. perfringens* strains formed a distinct, closely related group based on their *plc* gene sequences. These Egyptian strains were more closely related to the Japanese strains, with bootstrap support values of 59%. Among the C. *perfringens* sequences, MN635790 and MN635792 were the most divergent. The branching order within the other strains was highly reliable, indicated by high bootstrap values. Interestingly, MN635790 and MN635792 formed a unique cluster distinct to D63911 (Japan). These findings align with the only available complete *plc* gene sequence data on GenBank.

#### Diversity matrix of phospholipase C partial CDS

The identity matrix Table (5) shows that the detected phospholipase C sequences achieved high identity percentages (> 99%) for all isolates except strain PP002324. This strain displayed a significantly lower identity score (34.1%) compared to the other strains in the study. Notably, the selected strains represent diverse geographical locations worldwide, including Brazil, China, Denmark, England, Japan, Kazakhstan, India, Saudi Arabia, and the USA.

**Table 5:**
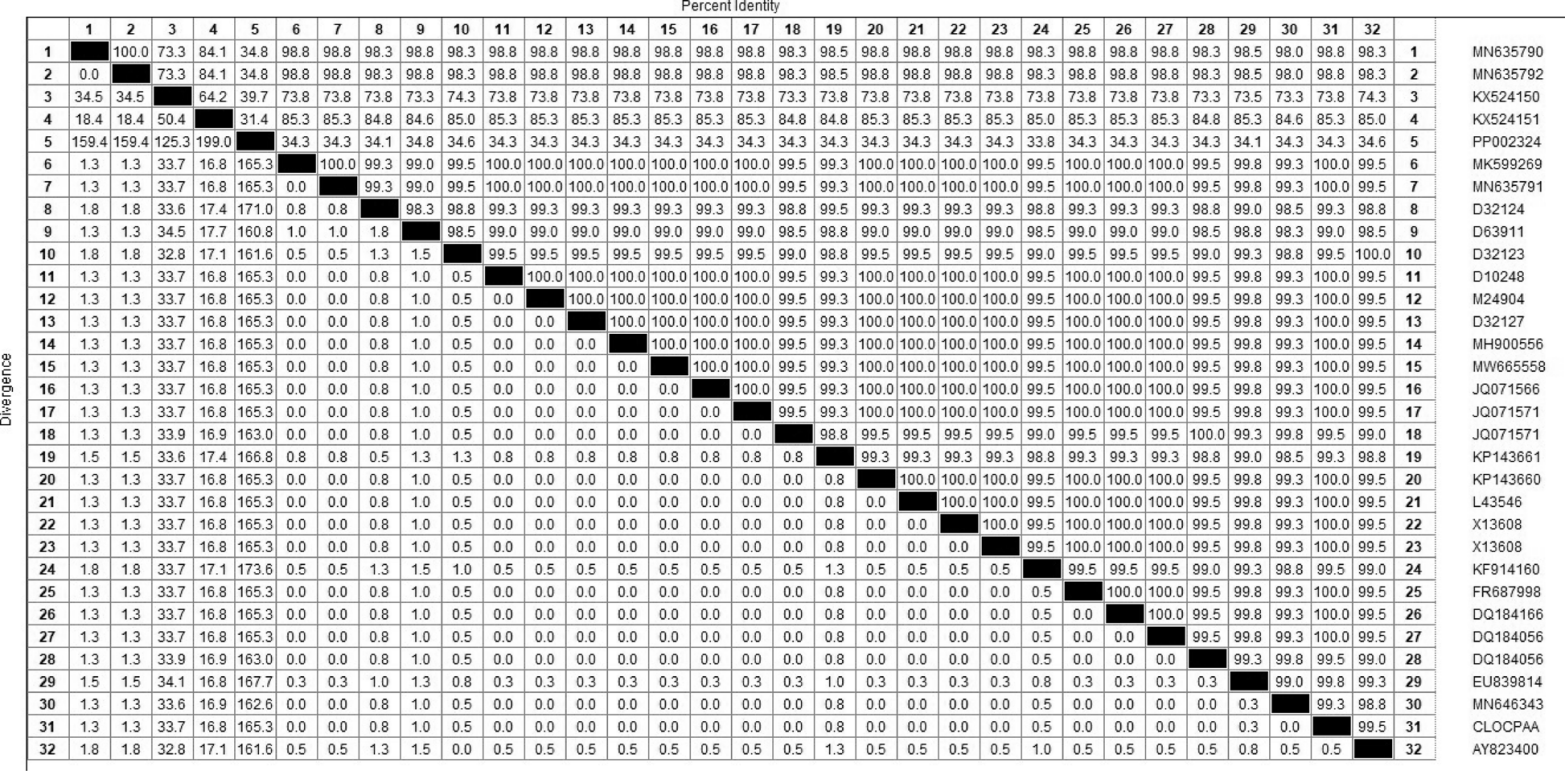
The divergence and identity matrix between selected five plc *partial CDS* of C. perfringens

The ML topology showed three major clades with low bootstrap values. The most divergent strains PP002324 and KX524150 with a maximum divergence of (173.6%) & (199%), respectively, most of the Egyptian strains were localized in the first clade (A) (Fig. 5).

The comparative similarity matrix output of the MegAlign module of Lasergene DNAStar software Pairwise (Table 5) shows the highest degree of divergence between Egyptian isolates, mainly PP002324 (Intestinal content), KX524150 (bovine milk), KX524151(baby calf of sudden death case) and MN635792 (Baby calves feces). The divergence percentage between Egyptian strains is (159.4%,199% ,119.8%, and 159.4%).

The sequence of phospholipase C from *C. perfringens* was subjected to phylogenetic analysis with the nucleotide sequences of phospholipase C from different reference strains from different countries. According to the alignment report, the selected sequences represent countries from different continents to trace the main origin or ancestry of our Egyptian strains.

As shown in Fig. (5), three distinct clades were resolved (15%, 18%, and less than 9% bootstrapping scores) as seen from the phylogenetic analysis of their bootstrapping scores and amino acid sequence data. Phylogenetic tree analysis supports the inference of the identified signature that these groups do not share a homology clade one with a degree (18% for 1st clades). The (A) clade was subdivided into two sub-clades (1 A) & (2 A), including a homology between MN635790 and MN635792 (faecal samples from diarrheal baby calves), while the KX524151 (bovine feaces) and KX524150 (bovine milk). PP002314 bootstrapping support by 35% to other Egyptian bovine strains.

The Japanese strain (D63911) is closer to the Egyptian strain by a 99% bootstrap score. Following that, Kazakhstan strain (KP143661)(68%), Chinese strains (AY823400) and Japanese strains (D32132) (57%).

*Plc* region sequences obtained in this study were compared with similar sequences obtained from GenBank. The sequences from the Egyptian strains were grouped into only one clade (A), except strain KX524151 was phylogroup into clade (B).

Sixteen sequences were clustered into two groups (clades B, and C) together with some other sequences from GenBank (Fig. 5). The difference in sequence was not related to the pathogenicity of the strain, as all organisms investigated in this study were obtained from different sites and under different pathogenic conditions.

## Discussion

Egypt’s robust milk production contributes significantly to the livelihoods of rural communities and national well-being [26]. The data about *C. perfringens* based infection in dairy farms is limited. However, it is known that mastitis is a common problem in Egyptian dairy farms, leading to economic losses [12].

Mastitis is one of the most important problems in dairy cattle. *C. perfringens* type A infections pose a particular danger to postpartum dairy cows, potentially triggering aggressive mastitis with sudden symptoms and high fatality rates [27].

Most mastitis-infected dairy cows carried *C. perfringens* type A, but specific toxins linked to mastitis weren’t found. This suggests *C. perfringens* might contribute to uterine and mastitis issues, but more research is needed to confirm its role and identify the key factors involved [2, 28].

Since the beginning of Osman and his colleague’s work, there has been no reference data on the current situation of *C. perfringens* and mastitis cases in dairy farms in Egypt [12].

In the present work, the recovery results of *C. perfringens* have been demonstrated at a rate of 23.5% overall in examined samples from dairy farms. The occurrence of *C. perfringens* in milk samples was 9% suggesting the role of this pathogen in causing disease in dairy animals.

Despite the intensive vaccination programs on these dairy farms to control Clostridium infection, the recorded proportion of *C. perfringens* isolated in cases of mastitis is very high and alarming compared to what was identified in Belgian dairy cattle by [29].

On the other hand, clinical samples collected from diarrheal cases in baby calves, or intestinal contents in sudden death cases revealed an isolation rate of *C. perfringens* of 32.9% and 100%, respectively. The results maximize the role of circulating *C. perfringens* in dairy farm systems as a food-borne contaminant for lactating baby calves. Acute enteritis and fatal enterotoxemia in animals have been attributed to *C. perfringens*, and the pathogenicity of this organism is associated with enterotoxins [12, 27].

Toxin genotyping is considered a convenient and highly reliable tool for the molecular detection of all major toxin genes, such as (*cpa*), (*cpb*1), (*etx*), and (*iap*). The designed multiplex PCR was found to be a suitable tool for toxin genotyping of *C. perfringens* isolates and for detecting the presence of *cpe* in the tested isolates.

According to multiplex PCR results, the present study indicated that type A is the most frequently isolated genotype of *C. perfringens*. A total of 93 *C. perfringens* isolates were classified as type A toxin producers; all are enterotoxigenic strains (*cpe*^+ve^). However, those strains isolated from the sudden death cases were mainly of type A, except for only four strains that were of type C (4.3%) (Table 3).

Globally, about 5% of all *C. perfringens* isolates produce a toxin named *C. perfringens* enterotoxin (*cpe*) [30]. Most (*cpe*^+^) strains are classified as type A, although types C and D strains producing this enterotoxin are also common [31, 32].

These results were in agreement with 12, 13, and 27. Alpha toxin is produced at a high level in type A and is involved in the pathogenesis of various diseases in animals [16, 33]. It is the main lethal toxin of *C. perfringens*, a multifunctional phospholipase produced by almost all isolates. The toxin is hemolytic, necrotizing, and effectively lethal [34].

*C. perfringens* enterotoxin (CPE) is responsible for causing the gastrointestinal symptoms of several *C. perfringens* food-borne and non-food gastrointestinal diseases in humans. According to [35] the gene for CPE (*cpe*) is located either on the chromosomes of most *C. perfringens* type A food poisoning strains or on large conjugative plasmids of the remaining type A food poisoning and most, if not all, other CPE-producing strains.

Another theory stated that the enterotoxin CPE encoded by the *cpe* gene is the only toxin known to occur on both chromosomes and plasmids. Interestingly, *cpe* has been found as plasmid-borne in strains isolated from livestock or non-food-borne human gastrointestinal cases (i.e. antibiotic-associated diarrhea or sporadic diarrhea) [36, 37]. In addition, *cpe* is only released during the sporulation of *C. perfringens* [38].

However, previous research has found that dairy farms are not a significant source of cpe-positive isolates, which agrees with [39], who found a low recovery in ruminant-associated isolates (2.9%) and [40], who found a high incidence of *cpe* carriage among canine, equine, and food isolates with an incidence of 94.1%, 93.8%, and 86.7%, respectively.

There are limited studies on the analysis of the *cpe* sequence of strains of bovine origin, so, in this study, a primer set was designed for the amplification of the *plc* gene of five enterotoxogenic strains from two different sources of feces and milk.

Bovine necrohaemorrhagic enteritis caused by *C. perfringens* is an important cause of sudden death with necrohaemorrhagic lesions in the small intestine [28]. The disease frequently strikes calves without warning symptoms in good to excellent bodily health who are fed huge amounts of milk or milk substitute [41]. Although mortality is very close to 100%, the disease has a significant economic impact despite the relatively low morbidity. From this fact, we apply molecular sequencing for the *plc* region from *C. perfringens* strains of lactating animals, diarrheal calves, and sudden death cases to investigate the pathogenesis of enterotoxigenic strains in dairy farming chains.

Many studies have sequenced the *cpe* gene [42, 43]. In the present work, the *plc* gene was amplified by novel oligonucleotide primers and sequenced for five strains. The nucleotide sequences of the selected Phospholipase C amino acid sequences have main regions of identity with other queries of different *C. perfringens* isolates. The detected complete phospholipase C sequences (MN635790 & MN635792 ) are not completely identical for different isolates with maximum query coverage (Fig. 5).

**Fig. 6:**
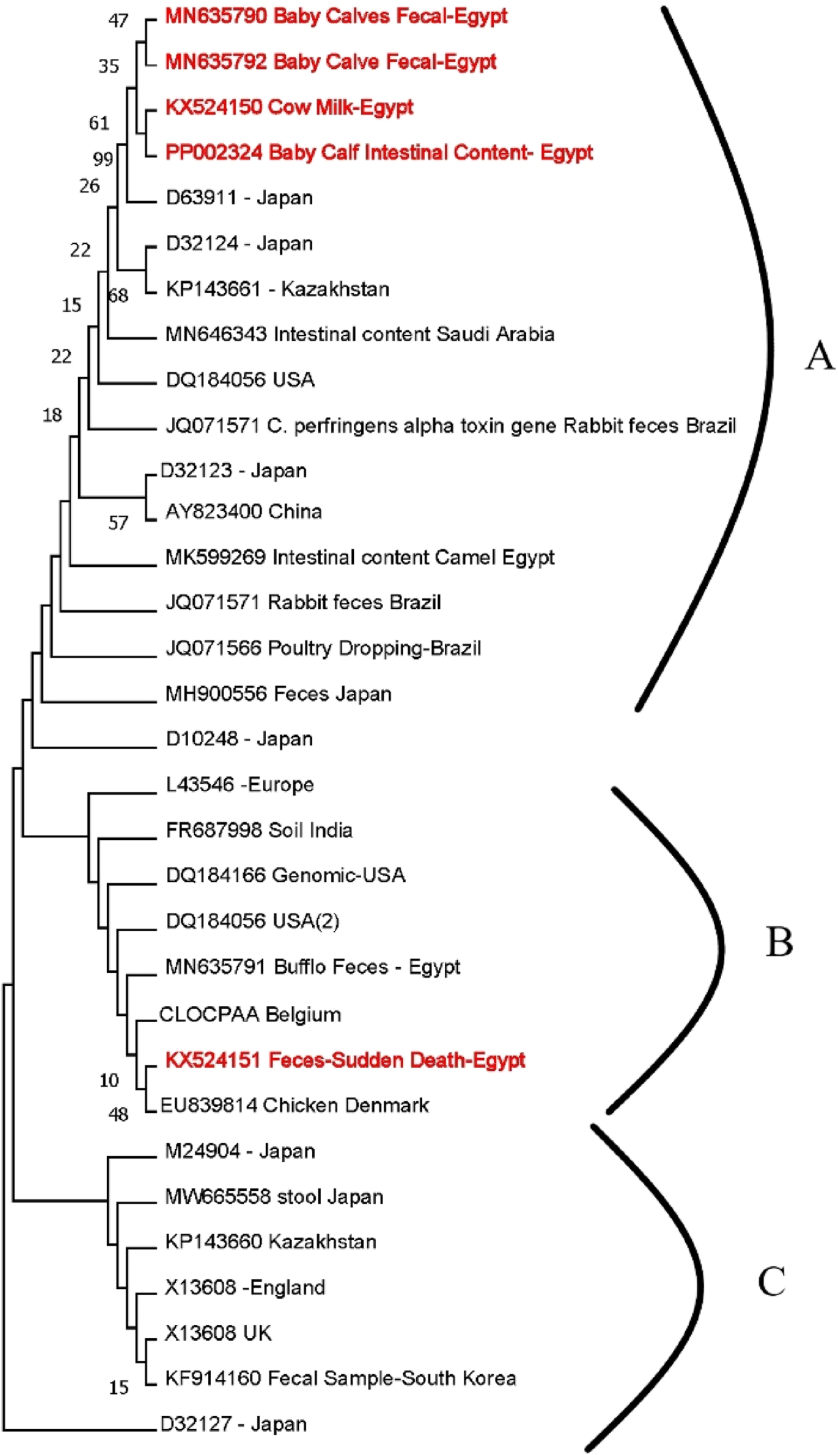
The evolutionary tree for *plc* gene partial CDS *C. perfringens* isolates and the obtained 27 referral nucleotide sequences from GenBank using the Maximum Parsimony method. The bootstrap consensus tree was inferred from 1000 replicates This analysis involved 22 nucleotide sequences. There were a total of 408 positions in the final dataset. Evolutionary analyses were conducted in MEGA11. Red labelled strains (The Egyptian strains in the present study)

That result of partial CDS sequence for *plc* gene confirms the complete homology of the milk and diarrheal strains, which subsequently related to a neco-hemorrhagic lesion of the intestine (Fig. 6). The results can be confirmed by the role of *C. perfringens* type A in clostridial abomasitis, which was confirmed when intraluminal administration of *C. perfringens* type A to neonatal calves induced clinical signs similar to naturally acquired disease [44]. *C. perfringens* type A strains were isolated almost exclusively from animals diagnosed with either necro-hemorrhagic enteritis [7, 45] or clostridial abomasitis [46, 47].

The enterotoxin (cpe), which acts as a warning to food-posing strains in humans, plays a significant role in the development of intestinal sickness in many animal species, including human. In contrast to CPE-associated non-food-borne human gastrointestinal disorders, it has been shown that the majority, if not all, *C. perfringens* type A food poisoning isolates carry a plasmid-based copy of the *cpe* gene [36, 37].

House et al. 2014 [47] reported that *C. perfringens* type C can cause sudden death in neonatal calves less than 10 days old, which is completely confirmed with the current data as four isolates of sudden death cases harbored type C toxin genes. The recovery rate of type C was 4.3%, which is extremely high compared to the early work of Omer et al. 2020 [43] in Saudia Arabia with the percentage of type C records reaching 0.96%. Although several textbooks describe the occurrence of *C. perfringens* type C infection in calves worldwide [48, 49].

Neonates can pick up type C bacteria from a habitat that has been contaminated by sick animals or, less frequently, by asymptomatic carriers. CPB is incredibly sensitive to trypsin and other protease activity. Newborn animals suffer from a protease deficiency in the intestine layers and absorption into the system. Death may be caused directly by severe intestinal necrosis and diarrhea, indirectly by subsequent toxemia, or both directly and indirectly.

## Conclusion

The present data conclusively confirms the role of *C. perfringens* type A in bovine necro-hemorrhagic enteritis and mastitis. First, it definitively demonstrates that the present understanding of intestinal disorders and mastitis associated with *C. perfringens*, which mostly focuses on chromosome-borne, disease-specific toxins, is overly restrictive and that alpha toxin can be important.

The discovery that *C. perfringens* type C is necessary for the development of necrotizing enteritis has major ramifications for immunization tactics. This helps to explain why the present clostridial vaccines, which are based on formaldehyde-inactivated toxins, don’t seem to protect against intestinal illnesses in calves caused by *C. perfringens* type A. New vaccinations are required to protect animals from gastrointestinal diseases associated with *C. perfrin*gens type A and C.

## Data Availability

The data presented in this study are deposited in NCBI (accession numbers: KX524150-: KX524151-MN635790- PP002324 and MN635792). Further inquiries can be directed to the corresponding author.

## Abbreviations

Cpa: Alpha toxin gene
SDS: Sudden Death Syndrome
CPE: *C. perfringens *enterotoxin

## References

[1] Dos Santos RAN, Abdel-Nour J, McAuley C, Moore SC, Fegan N, Fox EM (2022). *Clostridium perfringens* associated with dairy farm systems show diverse genotypes. Int J Food Microbiol.

[2] Grenda T, Jarosz A, Sapała M, Grenda A, Patyra E, Kwiatek K (2023). *Clostridium perfringens*—opportunistic Foodborne Pathogen, its diversity and epidemiological significance. Pathogens.

[3] Lugli GA, Milani C, Mancabelli L, Turroni F, Ferrario C, Duranti S, van Sinderen D, Ventura M (2017). Ancient bacteria of the Ötzi’s microbiome: a genomic tale from the copper age. Microbiome..

[4] Milton AAP, Momin KM, Priya GB, Ghatak S, Gandhale PN, Angappan M, Das S, Sen A (2021). A novel in situ methodology for visual detection of *Clostridium perfringens* in pork harnessing saltatory rolling circle amplification. Anaerobe.

[5] Anju K, Karthik K, Divya V, Mala Priyadharshini ML, Sharma RK, Manoharan S (2021). Toxinotyping and molecular characterization of antimicrobial resistance in *Clostridium perfringens* isolated from different sources of livestock and poultry. Anaerobe.

[6] McClane BA, Uzal FA, Miyakawa MF, Lyerly D, Wilkins TD, Dworkin M, Falkow S, Rosenburg E, Schleifer H, Stackebrandt E (2006). The enterotoxic clostridia. The prokaryotes.

[7] Manteca C, Daube G, Jauniaux T, Linden A, Pirson V, Detilleux J, Ginter A, Coppe P, Kaeckenbeeck A, Mainil JG (2002). A role for the *Clostridium perfringens* beta2 toxin in bovine enterotoxaemia?. Vet Microbiol.

[8] Miserez R, Frey J, Buogo C, Capaul S, Tontis A, Burnens A, Nicolet J (1998). Detection of alpha- and epsilon-toxigenic *Clostridium perfringens* type D in sheep and goats using a DNA amplification technique (PCR). Lett Appl Microbiol.

[9] Songer JG (1996). Clostridial enteric diseases of domestic animals. Clin Microbiol Rev.

[10] Grass JE, Gould LH, Mahon BE (2013). Epidemiology of food borne disease outbreaks caused by *Clostridium perfringens*, United States, 1998–2010. Foodborne Pathog Dis.

[11] Uzal FA, Freedman JC, Shrestha A, Theoret JR, Garcia J, Awad MM, Adams V, Moore RJ, Rood JI, McClane BA (2014). Towards an understanding of the role of *Clostridium perfringens* toxins in human and animal disease. Future Microbiol.

[12] Osman KM, El-Enbaawy MI, Ezzeldeen NA, Hussein HM (2009). Mastitis in dairy buffalo and cattle in Egypt due to *Clostridium perfringens* : prevalence, incidence, risk factors and costs. Rev Sci Tech.

[13] Ginter A, Williamson ED, Dessy F, Coppe P, Bullifent H, Howells A, Titball RW (1996). Molecular variation between the alpha-toxins from the type strain (NCTC 8237) and clinical isolates of *Clostridium perfringens* associated with disease in man and animals. Microbiol (Reading).

[14] Johansson A, Aspan A, Bagge E, Båverud V, Engström BE, Johansson KE (2006). Genetic diversity of *Clostridium perfringens* type a isolates from animals, food poisoning outbreaks and sludge. BMC Microbiol.

[15] Abildgaard L, Schramm A, Rudi K, Højberg O (2009). Dynamics of Plc gene transcription and alpha-toxin production during growth of *Clostridium perfringens* strains with contrasting alpha-toxin production. Vet Microbiol.

[16] Hamza D, Dorgham SM, Elhariri M, Elhelw R, Ismael E (2018). New insight of apparently healthy animals as a potential Reservoir for *Clostridium perfringens* : a Public Health Implication. J Vet Res.

[17] Hill JE, Penny SL, Crowell KG, Goh SH, Hemmingsen SM (2004). cpnDB: a chaperonin sequence database. Genome Res.

[18] Hirvonen J, Pyörälä S, Heinäsuo A, Jousimies-Somer H (1994). Penicillin G and penicillin G-tinidazole treatment of experimentally induced summer mastitis–effect on elimination rates of bacteria and outcome of the disease. Vet Microbiol.

[19] Murray PR, Baron EJO, Jorgensen JH, Pfaller MA, Yolken RH (2003). Manual of clinical microbiology.

[20] van Asten AJ, Nikolaou GN, Gröne A (2010). The occurrence of cpb2-toxigenic *Clostridium perfringens* and the possible role of the beta2-toxin in enteric disease of domestic animals, wild animals and humans. Vet J.

[21] Meer RR, Songer JG (1997). Multiplex polymerase chain reaction assay for genotyping *Clostridium perfringens*. Am J Vet Res.

[22] Altschul SF, Madden TL, Schäffer AA, Zhang J, Zhang Z, Miller W, Lipman DJ (1997). Gapped BLAST and PSI-BLAST: a new generation of protein database search programs. Nucleic Acids Res.

[23] Hall TA (1999). BioEdit: a user-friendly biological sequence alignment editor and analysis program for Windows 95/98/NT. Nucleic Acids Symp Ser.

[24] Sneath PHA, Sokal RR (1973). Numerical Taxonomy - The Principle and Practice of Numerical Classification.

[25] Thompson JD, Higgins DG, Gibson TJ (1994). CLUSTAL W: improving the sensitivity of progressive multiple sequence alignment through sequence weighting, position-specific gap penalties and weight matrix choice. Nucleic Acids Res.

[26] FAOSTAT. Developing the Dairy Value Chain in Egypt’s Delta in FAOSTAT. 2016. ISBN: 9789220327623.

[27] Lu W, Sun H, Xu ZM, Du Z, Si L, Yuan S, Jin J, Jin CH (2022). Diagnostic and therapeutic strategy for Clostridium perfringens infection in postpartum dairy cows: a report of 14 cases. J Appl Anim Res.

[28] Kronfeld H, Kemper N, Hölzel C (2022). Phenotypic and genotypic characterization of C. Perfringens isolates from dairy cows with a pathological puerperium. Vet Sci.

[29] Goossens E, Valgaeren BR, Pardon B, Haesebrouck F, Ducatelle R, Deprez PR, Van Immerseel F (2017). Rethinking the role of alpha toxin in *Clostridium perfringens* -associated enteric diseases: a review on bovine necro-haemorrhagic enteritis. Vet Res.

[30] Manteca C, Daube G, Pirson V, Limbourg B, Kaeckenbeeck A, Mainil JG (2001). Bacterial intestinal flora associated with enterotoxaemia in Belgian blue calves. Vet Microbiol.

[31] Kokai-Kun JF, Songer JG, Czeczulin JR, Chen F, McClane BA (1994). Comparison of western immunoblots and gene detection assays for identification of potentially enterotoxigenic isolates of *Clostridium perfringens*. J Clin Microbiol.

[32] Li J, Miyamoto K, Sayeed S, McClane BA (2010). Organization of the cpe locus in CPE-positive *Clostridium perfringens* type C and D isolates. PLoS ONE.

[33] Das A, Mazumder Y, Dutta BK, Shome BR, Bujarbaruah KM (2012). Molecular typing of *Clostridium perfringens* isolated from diarrhoeic cattle. J Anim Sci Adv.

[34] Petit L, Gibert M, Popoff MR (1999). *Clostridium perfringens* : toxinotype and genotype. Trends Microbiol.

[35] Freedman JC, Shrestha A, McClane BA (2016). *Clostridium perfringens* Enterotoxin: Action, Genetics, and translational applications. Toxins (Basel).

[36] Cornillot E, Saint-Joanis B, Daube G, Katayama S, Granum PE, Canard B, Cole ST (1995). The enterotoxin gene (cpe) of *Clostridium perfringens* can be chromosomal or plasmid-borne. Mol Microbiol.

[37] Collie RE, McClane BA (1998). Evidence that the enterotoxin gene can be episomal in *Clostridium perfringens* isolates associated with non-food-borne human gastrointestinal diseases. J Clin Microbiol.

[38] Duncan CL (1973). Time of enterotoxin formation and release during sporulation of *Clostridium perfringens* type A. J Bacteriol.

[39] Geier RR, Rehberger TG, Smith AH (2021). Comparative Genomics of *Clostridium perfringens* reveals patterns of host-Associated phylogenetic clades and virulence factors. Front Microbiol.

[40] Fohler S, Klein G, Hoedemaker M, Scheu T, Seyboldt C, Campe A, Jensen KC, Abdulmawjood A (2016). Diversity of *Clostridium perfringens* toxin-genotypes from dairy farms. BMC Microbiol.

[41] Lebrun M, Mainil JG, Linden A (2010). Cattle enterotoxaemia and *Clostridium perfringens* : description, diagnosis and prophylaxis. Vet Rec.

[42] Elgioushy M, Rizk MA, El-Adl M, Elhadidy M, El-Khodery S (2019). The first molecular detection of *Clostridium perfringens* from pneumonic cases associated with foot and mouth disease in cattle and buffalo in Egypt. Trop Anim Health Prod.

[43] Omer SA, Al-Olayan EM, Babiker SEH, Aljulaifi MZ, Alagaili AN, Mohammed OB (2020). Genotyping of *Clostridium perfringens* isolates from domestic livestock in Saudi Arabia. Biomed Res Int.

[44] Roeder BL, Chengappa MM, Nagaraja TG, Avery TB, Kennedy GA (1988). Experimental induction of abdominal tympany, abomasitis, and abomasal ulceration by intraruminal inoculation of *Clostridium perfringens* type A in neonatal calves. Am J Vet Res.

[45] Daube G, Simon P, Limbourg B, Manteca C, Mainil J, Kaeckenbeeck A. Hybridization of 2,659 Clostridium perfringens isolates with gene probes for seven toxins (alpha, beta, epsilon, iota, theta, mu, and enterotoxin) and for sialidase. Am J Vet Res. 1996 ;57(4):496-501. PMID: 8712513. 8712513

[46] Glenn Songer J, Miskimins DW (2005). Clostridial abomasitis in calves: case report and review of the literature. Anaerobe.

[47] House JK, Smith GW, McGuirk SM, Gunn AA, Izzo M, Smith BP (2014). Manifestations and management of disease in neonatal ruminants. Large animal internal medicine.

[48] Brown CC, Baker DC (2007). Baker IK alimentary system. Jubb, Kennedy & Palmer’s pathology of domestic animals.

[49] Gelberg HB, Zachary JF, McGavin MD (2007). Alimentary system. Pathologic basis of veterinary disease.

